# CSK-DDI: compensating structural incompleteness with knowledge graph world knowledge for drug–drug interaction modeling

**DOI:** 10.1093/bioinformatics/btag800

**Published:** 2026-10-28

**Authors:** Jiayi Wang, Yuqin He, Tengfei Ma, Bosheng Song

**Affiliations:** College of Computer Science and Electronic Engineering, Hunan University, Changsha, Hunan 410082, China; College of Computer Science and Electronic Engineering, Hunan University, Changsha, Hunan 410082, China; College of Computer Science and Electronic Engineering, Hunan University, Changsha, Hunan 410082, China; College of Computer Science and Electronic Engineering, Hunan University, Changsha, Hunan 410082, China; Yuelushan Laboratory, Changsha, Hunan 410128, China

## Abstract

**Motivation:**

Drug–drug interaction (DDI) prediction is critical for drug safety, yet existing methods suffer from structural incompleteness in drug representation. Motif and molecular graph encodings capture local substructures and global topology but remain confined to the closed structural space of individual molecules, lacking cross-entity and contextual information and leading to limited generalization, especially in cold-start scenarios. To address this issue, we propose CSK-DDI (Compensating Structural Incompleteness with Knowledge Graph World Knowledge for Drug–Drug Interaction Modeling), a multimodal framework that jointly encodes motif-level chemical patterns, molecular graph topology, and biomedical knowledge graph (KG) context as complementary drug representations. The KG encodes drugs’ contextual positions and relational semantics through multi-entity and multi-relation connections, providing global cross-entity and cross-semantic information that complements structural representations. The resulting fusion captures local chemical patterns, global topology, and biomedical context in a unified embedding.

**Results:**

CSK-DDI employs a cross-attention mechanism to enable multi-view interaction among motif, molecular graph, and KG representations, producing unified structure-aware and context-aware drug embeddings. To improve robustness under sparse conditions, the model further incorporates a cold-start-oriented representation enhancement strategy. Experiments on benchmark datasets demonstrate consistent improvements over state-of-the-art methods, with particularly notable gains in cold-start scenarios.

**Availability and implementation:**

Source code and data are available at https://github.com/cgwcbz2/CSK-DDI and on Zenodo at https://doi.org/10.5281/zenodo.21415769.

## 1 Introduction

With the increasing prevalence of complex diseases and population aging, combination therapy has become more common (Fisusi and Akala 2019, Jaaks *et al.* 2022). While drug combinations can expand treatment options and improve therapeutic efficacy (Lim 2020, Khunsriraksakul *et al.* 2022), they may also induce adverse drug reactions caused by drug–drug interactions (DDIs; Lyu *et al.* 2021, Jiang *et al.* 2022), leading to increased morbidity and mortality (Chen *et al.* 2021). Therefore, early prediction of potential DDIs is crucial for ensuring patient safety in clinical practice.

Traditional experimental methods for DDI identification and prediction are costly and time-consuming, making large-scale DDI discovery challenging (Yu *et al.* 2018). DeepDDI (Ryu *et al.* 2018) predicted potential DDIs using only drug names and SMILES representations. Given that molecular motifs are key structural units underlying drug activity and pharmacological properties, some studies have incorporated substructure features. To integrate diverse drug characteristics, the drug-drug interaction multimodal deep learning (DDIMDL) framework (Deng *et al.* 2020) jointly learned cross-modal representations from chemical substructures, targets, enzymes, and pathways. The ChemicAl SubstrucTurE Representation (CASTER) framework (Huang *et al.* 2020) improved DDI interpretability by identifying interaction-related substructures through a dictionary learning module.

These methods represent drugs as discrete features or flat vectors, which limits their ability to capture global molecular topology and long-range dependencies. To address these limitations, graph neural networks (GNNs) have been introduced to model drugs as molecular graphs, capturing global structures via atom-bond message passing. Chen *et al.* (2019) employed graph convolutional networks (GCNs) to learn low-dimensional molecular representations for DDI prediction, while the multi-view graph contrastive representation learning framework for drug-drug interaction prediction (MIRACLE) (Wang *et al.* 2021b) further captured both inter-molecular structural relations across views and intra-view drug interactions. Furthermore, the multi-relational contrastive learning graph neural network (MRCGNN) (Xiong *et al.* 2023) integrated drug molecular graphs with a multi-relational DDI event graph via contrastive learning to effectively capture both structural and implicit interactive information.

Molecular graphs capture topological structure, but single representations may miss other molecular properties. The multi-type feature fusion based on graph neural network (MFFGNN) model (He *et al.* 2022) fused simplified molecular input line entry system (SMILES) sequences and molecular graphs by capturing local chemical semantics with BiGRU and structural features via graph interaction networks.

Recurrent networks and conventional GNNs face computational bottlenecks with complex molecules. The Transformer (Vaswani *et al.* 2017) architecture, leveraging global attention, provided a new paradigm to overcome these challenges. ChemBERTa (Chithrananda *et al.* 2020) showed that pre-trained Transformers can learn rich chemical semantics and syntax from large-scale SMILES corpora, and Graphormer (Ying *et al.* 2021) further improves topological awareness by integrating centrality and spatial encodings into attention, achieving superior molecular property prediction. To explicitly capture functional substructures related to DDIs, ImageDDI (He *et al.* 2026) employed a Transformer-based encoder to model motif sequences and fuse them with molecular features, while the dual-drug visual representation for drug-drug interaction prediction (DDVR-DDI) framework (Xie *et al.* 2025) adopts molecular images with a visual encoder and self-supervised pretraining for DDI prediction.

However, even though Transformers can effectively model internal drug structures, these methods remain confined to a closed molecular space. In practice, drug pharmacological activity depends not only on chemical structure but also on external relational information within biological networks. To integrate such multi-source information, knowledge graphs (KGs) have been introduced to provide rich biological context. KGs encode relationships between drugs and other biological entities as structured triples, complementing purely structural representations. KGNN (Lin *et al.* 2020) is an end-to-end framework that aggregates neighborhood information from KGs to capture high-order structural and semantic relations. The knowledge graph-enhanced multi-task learning (KG-MTL) framework (Ma *et al.* 2022) fuses KG and molecular graph features to learn comprehensive drug representations under a multi-task learning framework. The multi-scale feature fusion for drug–drug interaction prediction (MUFFIN) framework (Chen *et al.* 2021) fused molecular structures and KG semantics via a dual cross-layer strategy, while DeepLGF (Ren *et al.* 2022) improved DDI prediction by integrating local and global information from biomedical KGs. The Transformer-based relatIon-aware Graph rEpresentation leaRning (TIGER) framework (Su *et al.* 2024) jointly encoded drug molecular graphs and biomedical KGs to leverage both chemical topology and external biomedical context.

Nevertheless, most KG-based methods rely on pre-trained embeddings or shallow GNNs and fail to exploit Graph Transformers to deeply model structured information and long-range dependencies in biological networks, limiting the expressiveness of external contextual representations. Prior methods predominantly encode individual drug representations without incorporating external relational context that connects drugs within biological networks.

In this study, we propose a DDI prediction framework that unifies microscopic, macroscopic, and external contextual information. The model employs a Transformer to encode motif sequences for capturing fine-grained physicochemical semantics, and graph Transformers to extract global topological features from molecular graphs and external biological context from KG subgraphs. A cross-attention mechanism is then introduced to adaptively fuse these heterogeneous representations, producing comprehensive drug embeddings for DDI prediction. Extensive experiments on benchmark datasets demonstrate that the proposed model effectively integrates multi-source information and outperforms state-of-the-art methods in prediction accuracy.

The main contributions of this article are summarized as follows:

- We propose CSK-DDI, a multimodal framework that jointly encodes functional motifs, molecular graphs, and biomedical KGs to capture complementary drug interaction signals.
- We introduce a cross-attention-based fusion strategy to adaptively integrate heterogeneous drug representations from complementary chemical and biomedical modalities.
- Experiments demonstrate that CSK-DDI outperforms state-of-the-art methods, exhibits robust generalization in cold-start scenarios, and provides mechanistic interpretability.

## 2 Materials and methods

### 2.1 Notations

Definition 1 (motif sequence). Each drug d∈D is associated with a discrete motif sequence $S_{d}=\{s_{1},s_{2},\ldots ,s_{L}\}$, where each motif $s_{\ell }$ is a functional substructure unit belonging to a predefined motif vocabulary V.

Definition 2 (molecular graph). Each drug d∈D is represented as a molecular graph $G_{d}=(V_{d},E_{d})$, where $V_{d}$ denotes the set of atoms and $E_{d}$ denotes the set of chemical bonds. Each atom $v\in V_{d}$ is associated with a feature vector $x_{v}$ encoding intrinsic atomic properties. Each bond $(u,v)\in E_{d}$ between atoms $u,v\in V_{d}$ is associated with a bond type $r_{uv}\in \{1,\ldots ,R_{\text{mol}}\}$.

Definition 3 (KG). The external biomedical KG is defined as $G_{\text{kg}}=(E,R,T)$, where E denotes the set of entities, R denotes the set of relation types, and T denotes the set of triples. Entities are associated with feature vectors encoding biomedical properties, and triples are associated with relation types $r\in \{1,\ldots ,R_{\text{kg}}\}$. We assume D⊆E.

### 2.2 Problem formulation

Given a labeled dataset $T=\{(d_{i},d_{j},y_{ij})|d_{i},d_{j}\in D,y_{ij}\in C\}$, where C={1,…,C} denotes the set of interaction types, the goal of multi-class DDI prediction is to learn a mapping function $f_{\theta }:D\times D\to C$. This function predicts the interaction type $y_{ij}$ for any arbitrary drug pair $\left(d_{i},d_{j}\right)$ by jointly leveraging their motif sequences $S_{d}$, molecular graphs $G_{d}$, and the contextual information from the external KG $G_{\text{kg}}$.

### 2.3 Overview of CSK-DDI method

The overall architecture of the proposed CSK-DDI framework is illustrated in Fig. 1. The preprocessed multi-source inputs are first fed into feature extraction encoders. Specifically, discrete motif sequences are processed by a standard Transformer to capture motif features. Concurrently, molecular graphs and local subgraphs within KG augmented with virtual edges encoding shortest paths between nodes are passed through Relation-aware Graph Transformers to capture structural and biomedical contexts. These multimodal features are then combined via a cross-attention fusion mechanism, which aligns molecular and knowledge representations to address structural incompleteness. Finally, the fused representations of the interacting drug pair are concatenated and fed into a Multi-Layer Perceptron (MLP) to predict the DDI type.

**Figure 1:**
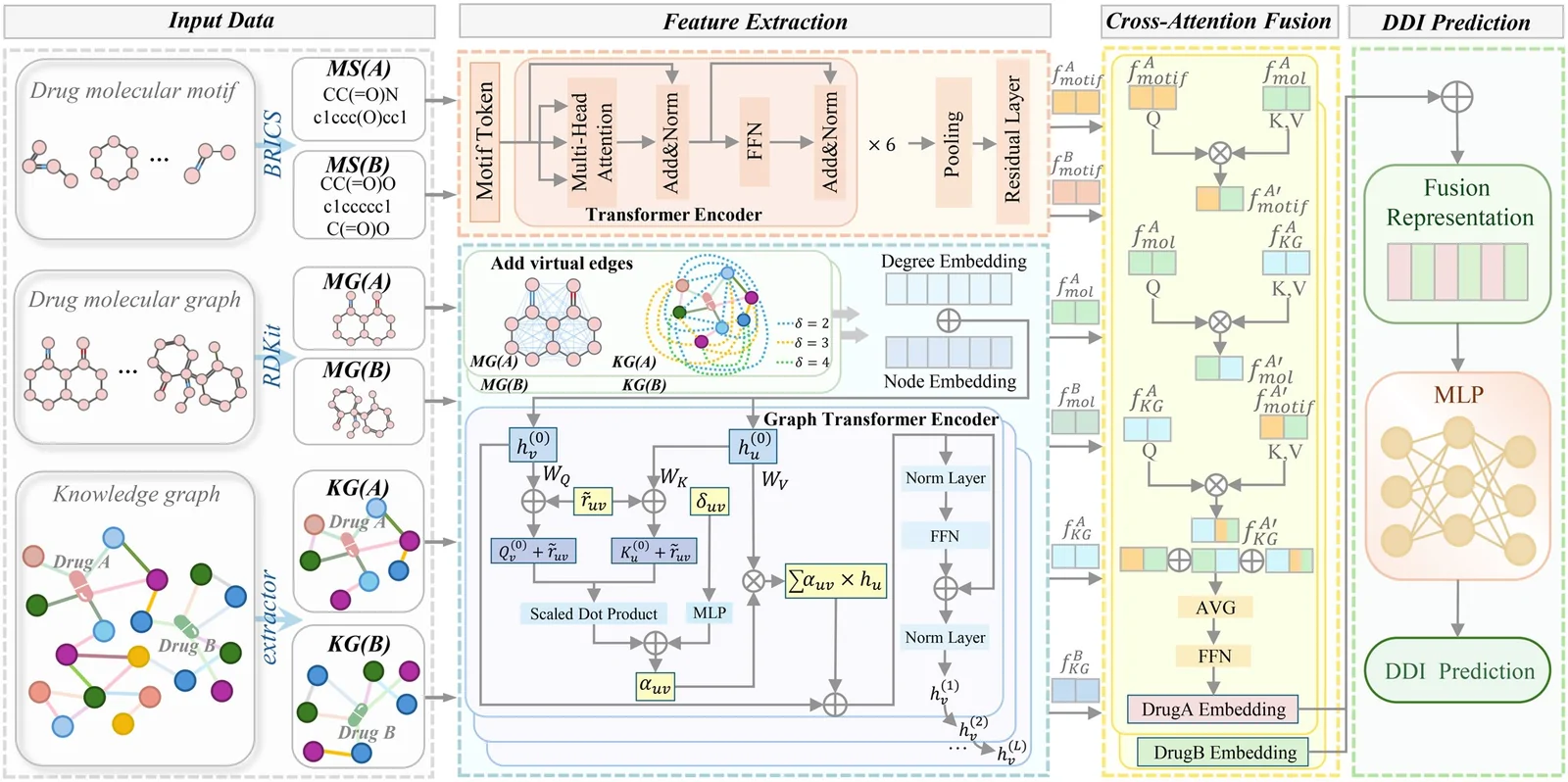
The overall architecture of the proposed CSK-DDI framework. (a) Each drug is represented by three modalities, including a motif sequence derived from SMILES, a molecular graph, and a localized subgraph from an external biomedical KG. (b) For feature extraction, a standard Transformer encodes motif sequences, while both molecular graphs and KG subgraphs are augmented with shortest-path virtual edges and then processed by relation-aware graph Transformers. (c) Cross-attention aligns and fuses heterogeneous features across modalities to form a unified drug representation. (d) The fused representations of a drug pair are concatenated and fed into a MLP for multi-class DDI prediction.

### 2.4 Feature extraction module

To model the heterogeneous properties of interacting drugs, the feature extraction module independently processes the multi-source data to generate modality-specific representations. Specifically, for a given drug pair $\left(d_{i},d_{j}\right)$, the module outputs three pairs of continuous feature vectors: motif representations $\left(f_{\text{motif}}^{d_{i}},f_{\text{motif}}^{d_{j}}\right)$, molecular graph representations $\left(f_{\text{mol}}^{d_{i}},f_{\text{mol}}^{d_{j}}\right)$, and biomedical context representations $\left(f_{\text{KG}}^{d_{i}},f_{\text{KG}}^{d_{j}}\right)$. The detailed encoding mechanisms for each modality are presented below.

#### 2.4.1 Transformer-based motif encoding

Molecular motifs represent functional substructures within molecules. Compared with atom-level representations, motif-level encoding provides a higher-level abstraction while reducing structural redundancy.

For each drug $d\in \{d_{i},d_{j}\}$, its SMILES string is decomposed into a motif sequence $S_{d}=\{s_{1},s_{2},\ldots ,s_{L}\}$ according to Definition 1. The motif vocabulary V is pre-constructed using the BRICS algorithm (Degen *et al.* 2008) to serve as a fixed structural fragment lexicon, as it fragments molecules at chemically meaningful bonds, preserving functional substructures such as rings and pharmacophoric groups that likely underlie DDI mechanisms. To capture contextual interactions and long-range dependencies within a molecule, we process the motif sequence using a standard Transformer encoder. The attention weight $\alpha_{km}$ between the *k*-th and *m*-th motifs in the self-attention mechanism is computed as:


(1) $$\alpha_{km}=\text{softmax}\left(\frac{{\left(W_{Q}h_{k}\right)}^{⊤}(W_{K}h_{m})}{\sqrt{d_{\text{head}}}}\right)$$

where $W_{Q}$ and $W_{K}$ are learnable projections, $d_{\text{head}}$ is the attention head dimensionality, and $h_{k}$ and $h_{m}$ denote the hidden states of the *k*-th and *m*-th motifs.

The resulting motif embeddings are aggregated via global mean pooling to obtain a sequence-level vector $\bar{h}$. A residual projection layer then produces the final motif representation for drug *d*:


(2) $$f_{\text{motif}}^{d}=\text{ReLU}(W_{\text{proj}}\bar{h}+b_{\text{proj}})+W_{\text{res}}\bar{h}$$

#### 2.4.2 Relation-aware graph encoding

Both the internal molecular structures and the external biomedical knowledge are represented as graphs. To encode them while preserving their topological and semantic properties, we employ a unified Relation-aware Graph Transformer architecture.

**Node initialization.** For the molecular graph $G_{d}$, which is constructed from the SMILES string using RDKit, we initialize each atom’s representation by fusing its intrinsic atomic features $x_{v}$ with its degree embedding:


(3) $$h_{v}^{\left(0\right)}=W_{\text{atom}}x_{v}+E_{\text{deg}}[\text{deg}(v)]$$

For the external knowledge context, directly encoding the complete $G_{\text{kg}}$ is computationally expensive and may introduce irrelevant information. We therefore extract a localized drug-centered subgraph $G_{\text{sub}}$ by performing $N_{w}$ random walks of length $L_{w}$ starting from the target drug node *d*, collecting all visited entities as the subgraph nodes. The initial edges between these nodes are then induced directly from the original $G_{\text{kg}}$. Each entity node *e* in this subgraph is initialized by fusing a learnable entity embedding with its degree information:


(4) $$h_{e}^{\left(0\right)}=E_{\text{node}}[e]+E_{\text{deg}}[\text{deg}(e)]$$

**Relation-aware attention mechanism.** To capture long-range structural dependencies, both the molecular graphs and the extracted KG subgraphs are augmented with shortest-path virtual edges. For any reachable node pair (u,v) within the same graph with a shortest-path distance $\delta_{uv}>1$, a virtual edge is introduced. The augmented relation type ${\tilde{r}}_{uv}$ is formally defined as:


(5) $${\tilde{r}}_{uv}=\{\begin{array}{cc} r_{uv}, & \text{if}\;\delta_{uv}=1\\ R+\delta_{uv}, & \text{otherwise} \end{array}$$

where *R* denotes the total number of relation types in the corresponding original graph, specifically $R_{\text{mol}}$ for molecular graphs and $R_{\text{kg}}$ for KG subgraphs.

We employ a multi-layer Graph Transformer to propagate information across these augmented dense topologies. At each layer, the attention mechanism incorporates both the discrete relation embeddings and a continuous spatial bias:


(6) $$\alpha_{uv}\propto \;\text{exp}\;\left(\frac{{\left(q_{v}+e_{{\tilde{r}}_{uv}}\right)}^{⊤}(k_{u}+e_{{\tilde{r}}_{uv}})}{\sqrt{d_{\text{head}}}}+\text{MLP}(\delta_{uv})\right)$$

where $q_{v}$ and $k_{u}$ are the query and key vectors of the target node *v* and source node *u*, respectively. $e_{{\tilde{r}}_{uv}}$ is the learnable embedding of the augmented relation type ${\tilde{r}}_{uv}$, and $\text{MLP}(\delta_{uv})$ applies a learnable spatial decay based on the shortest-path distance. Node representations are iteratively updated through multi-head attention accompanied by residual connections and feed-forward networks.

**Graph readout.** After *L* layers of propagation, different readout strategies are applied based on the graph type. For the molecular graph, we apply global mean pooling over all atoms and project it via a residual layer to yield the molecular representation $f_{\text{mol}}^{d}$. For the KG subgraph, we directly extract the representation of the central target drug node to represent the biomedical context, yielding $f_{\text{KG}}^{d}$.

### 2.5 Cross-attention fusion module

To integrate the heterogeneous features from the molecular motif, molecular graph, and KG subgraph channels, we design a cross-attention fusion module. This module adaptively aligns information across different semantic spaces, ensuring that each representation is enriched by the context of the others. For each drug *d*, the initial feature set $\left\{f_{\text{motif}}^{d},f_{\text{mol}}^{d},f_{\;\text{KG}}^{d}\right\}$ undergoes a sequential cross-view refinement process.

The core mechanism employs multi-head attention with *H* heads and dropout rate *p*. For a source feature $f_{s}$ querying a target feature $f_{t}$, the cross-attention operation is defined as:


(7) $$\text{CrossAttn}(f_{s},f_{t})=\text{MultiheadAttn}(f_{s},f_{t},f_{t};H,p)$$

where the first argument acts as the query, and the second and third serve as keys and values, respectively.

The fusion process follows a consistent cross-modal refinement mechanism. For any two modalities with features $f_{A}$ and $f_{B}$, the update is formulated as:


(8) $$f_{A}^{\prime }=\text{LN}(f_{A}+\text{Attn}(f_{A},f_{B}))$$

where LN(·) and Attn(·) denote layer normalization and cross-attention, respectively.

This operation is applied sequentially across three stages: (i) motifs refined by graphs, (ii) graphs refined by the KG, and (iii) the KG refined by the updated motifs. This cycle ensures a closed-loop information update, bridging structural features with extrinsic pharmacological pathways. This sequence follows a progression from fine-grained local motifs to overall atomic topology and finally to external pharmacological context from the KG, before closing the loop back to further enrich the motif representation with this accumulated cross-modal information, thereby completing a coherent bottom-up refinement.

The final integrated drug representation $z_{d}$ is generated by aggregating the refined features from all three channels:


(9) $$z_{d}=\text{FFN}\left(\frac{f_{\text{motif}}^{\prime }+f_{\text{mol}}^{\prime }+f_{\text{KG}}^{\prime }}{3}\right)$$

where the feed-forward network $\text{FFN}(x)=W_{2}\text{ReLU}(W_{1}x)$ projects the averaged features through a hidden layer of dimension $d_{h}$, yielding a comprehensive drug representation $z_{d}\in R^{d}$ that captures intrinsic chemical attributes, atomic topology, and extrinsic biological environment.

### 2.6 DDI prediction module

For a given drug pair $\left(d_{i},d_{j}\right)$, the individual representations $z_{d_{i}}$ and $z_{d_{j}}$ are first generated via the multi-channel extraction and cross-attention fusion modules. These representations are then concatenated to form a joint drug-pair descriptor:


(10) $$z_{\text{pair}}=[z_{d_{i}}∥z_{d_{j}}]$$

where ∥ denotes the concatenation operation. This joint descriptor is fed into an MLP classifier consisting of $L_{\text{MLP}}$ layers with hidden dimension $d_{\text{MLP}}$. The classifier incorporates batch normalization and ReLU activation:


(11) $$h=\text{ReLU}\left(\text{BatchNorm}\left(W_{1}z_{\text{pair}}+b_{1}\right)\right)$$

(12) $$p=\text{Softmax}\left(W_{2}h+b_{2}\right)$$

where $p\in R^{C}$ represents the predicted probability distribution over *C* interaction types. The model is optimized end-to-end by minimizing the cross-entropy loss over the training dataset T:


(13) $$L=-\sum_{(d_{i},d_{j},y)\in T}\;\text{log}\;\;p[y]$$

where p[y] denotes the predicted probability for the ground-truth interaction type *y*. The model is trained using the Adam optimizer with learning rate η and weight decay (WD) λ, with a ReduceLROnPlateau scheduler to dynamically adjust η when validation loss plateaus.

## 3 Results

### 3.1 Experiment setup

#### 3.1.1 Datasets

We evaluate our model on three widely used benchmark datasets. (i) Deng’s dataset (Deng *et al.* 2020) contains 567 drugs and 37 159 interaction pairs across 65 event types. (ii) Ryu’s dataset (Ryu *et al.* 2018) comprises 1689 drugs and 191 075 pairs across 86 categories. Both are used for standard transductive evaluation. We further analyze the frequency distribution of DDI types in Deng’s and Ryu’s datasets by counting the number of interaction pairs for each DDI type. As shown in Table 1, both datasets exhibit long-tail distributions, with many DDI types containing limited interaction pairs. (iii) DrugBank dataset (Wishart *et al.* 2018) includes 1706 drugs and 191 808 pairs labeled with 86 interaction types. It is strictly partitioned for inductive evaluation, where S1 represents pairs with a single unseen drug and S2 denotes pairs where both drugs are unseen. Together, these three datasets provide complementary coverage of transductive and inductive DDI prediction, enabling a comprehensive evaluation of both interaction type classification among known drugs and generalization to previously unseen drugs.

**Table 1:** Distribution of DDI types according to interaction frequency.

Dataset	DDI event frequency			
	[1,50]	(50,100]	(1,00,300]	**(300,+** ∞ **)**
Deng’s	41.5%	24.6%	15.4%	18.5%
Ryu’s	26.7%	11.6%	14.0%	47.7%

Additionally, we employ the Drug Repurposing Knowledge Graph (DRKG; Ioannidis *et al.* 2020) as the external knowledge base, extracting drug-centered subgraphs to enrich representations with structured biological context. DRKG is a comprehensive biomedical KG containing 97 238 entities and 5 874 261 triplets, covering diverse biomedical entities, including drugs, genes, diseases, pathways, and biological processes. Its heterogeneous biomedical relations provide rich pharmacological context for drug-centered subgraph construction. Benchmark drugs are mapped to DRKG entities via DrugBank IDs, achieving a mapping success rate exceeding 99.6% across all datasets. The few unmapped drugs and their corresponding interaction pairs are excluded from KG-based subgraph construction and prediction.

#### 3.1.2 Baselines

We compare our model with several state-of-the-art baselines:

- DeepDDI (Ryu *et al.* 2018) extracts features from drug names and SMILES to directly classify interactions.
- SSI-DDI (Nyamabo *et al.* 2021) employs GAT on raw molecular graphs to capture micro-level substructure interactions.
- MRCGNN (Xiong *et al.* 2023) utilizes contrastive learning on dual-view features from molecular and DDI event graphs.
- KG-DDI (Kim and Cho 2019) integrates diverse biomedical attributes into entity embeddings using a hybrid CNN-LSTM.
- MUFFIN (Chen *et al.* 2021) jointly learns intrinsic drug structures and external KG semantics via a cross-level fusion strategy.
- GoGNN (Wang *et al.* 2021a) employs a dual-attention graph-of-graphs architecture to hierarchically extract features from both local structured entity graphs and the global interaction network.
- DDI-Hybrid (Ali *et al.* 2025) integrates convolutional and BiLSTM networks to capture local structural features and long-range dependencies from Morgan fingerprint-encoded structural similarity profiles.
- MI-DDI (Cheng *et al.* 2025) fuses atomic and substructure-view features through a dedicated interaction module to enable precise and interpretable DDI predictions.

To evaluate inductive generalization on unseen drugs, we further compare with knowledge-aware inductive baselines:

- CSMDDI (Liu *et al.* 2022) extracts relational knowledge from external biomedical networks via RESCAL tensor factorization.
- HIN-DDI (Tanvir *et al.* 2021) leverages meta-path analysis on a Heterogeneous Information Network for deductive prediction.
- DeepLGF (Ren *et al.* 2022) extracts and aggregates local-global topological information from a biomedical KG using SMILES.

#### 3.1.3 Metrics

We quantitatively assess predictive performance using standard metrics based on True Positives (*TP*), False Positives (*FP*), True Negatives (*TN*), and False Negatives (*FN*):

- Accuracy (ACC): provides the global correctness rate as $\frac{TP+TN}{TP+FP+FN+TN}$.
- Macro-precision (Macro-Pre.): balances false alarms by measuring positive prediction accuracy as $\frac{TP}{TP+FP}$ for each interaction class, and the reported Macro-Pre. is obtained by averaging the precision values across all classes.
- Macro-recall (Macro-Rec.): measures the ability to identify all true interactions as $\frac{TP}{TP+FN}$ for each interaction class, and the reported Macro-Rec. is obtained by averaging the recall values across all classes.
- Macro-F1: offers a balanced measurement via the harmonic mean as $\frac{2\times \mathrm{Precision}\times \mathrm{Recall}}{\mathrm{Precision}+\mathrm{Recall}}$ for each interaction class, and the reported Macro-F1 is obtained by averaging the F1-scores across all classes.

Under the standard transductive setting, we comprehensively report ACC, Macro-Pre., Macro-Rec., and Macro-F1. For the inductive cold-start setting, we evaluate the model using ACC and Macro-F1 as the primary performance indicators.

### 3.2 Implementation details

During the feature extraction and fusion phases, the hidden state dimension across all channels is uniformly set to $d_{h}=128$. A dropout rate of 0.2 is applied across the network layers to mitigate overfitting. The framework is optimized end-to-end using the Adam optimizer with an initial learning rate of 0.001 and a ReduceLROnPlateau scheduler to dynamically decay the learning rate when validation loss plateaus. For the KG subgraph extraction described, the number of random walks is set to $N_{w}=1$ with a walk length of $L_{w}=32$. The detailed configuration of parameters is provided in Table 1, available as supplementary data at *Bioinformatics* online.

Given the multi-class nature of the DDI prediction tasks, the model is optimized by minimizing the cross-entropy loss. Datasets are randomly partitioned into training, validation, and test sets at a ratio of 7:1:2. To assess inductive generalization, we formulate two cold-start scenarios: S1, where one drug in the test pair is unseen during training, and S2, where both drugs are entirely unseen.

### 3.3 Evaluation results

Table 2 summarizes the performance of CSK-DDI and comparative baselines on Deng’s and Ryu’s datasets in the transductive setting. Our proposed CSK-DDI demonstrates the most robust and superior performance across both datasets. Specifically, on Deng’s dataset, CSK-DDI outperforms the strongest baseline (MRCGNN) by margins of 1.35% on Accuracy, 0.73% on Macro-F1, 9.66% on Macro-Recall, and 0.83% on Macro-Precision. On the larger Ryu’s dataset, our model achieves the highest Accuracy (0.9716) and yields the best results across all other macro-level metrics.

**Table 2:** Performance evaluation in the transductive setting.^a^

Method	Deng’s dataset				Ryu’s dataset			
	ACC	Macro-F1	Macro-Rec.	Macro-Pre.	ACC	Macro-F1	Macro-Rec.	Macro-Pre.
DeepDDI	0.7807	0.6055	0.5839	0.6611	0.9323	0.8643	0.8512	0.8928
SSI-DDI	0.7866	0.4216	0.3896	0.5139	0.9008	0.6663	0.6287	0.7507
MRCGNN	0.8979	0.7791	0.7688	0.8101	0.9567	0.8894	0.8727	0.9221
KG-DDI	0.7876	0.5027	0.5109	0.5845	0.9121	0.7762	0.7867	0.8101
MUFFIN	0.8269	0.5245	0.4844	0.6204	0.9510	0.8566	0.8339	0.8980
GoGNN	0.8766	0.6938	0.6841	0.7316	0.9424	0.8589	0.8451	0.8949
DDI-Hybrid	0.8639	0.7162	0.6780	0.7923	0.9506	0.8536	0.8333	0.8921
MI-DDI	0.8559	0.6837	0.6649	0.7346	0.9473	0.8786	0.8549	0.9287
CSK-DDI-Concat	0.8815	0.7651	0.7595	0.8103	0.9594	0.8954	0.8955	0.9281
CSK-DDI-KM (w/Motif only)	0.8891	0.7293	0.7182	0.7657	0.9353	0.8764	0.8591	0.9145
CSK-DDI-KG (w/o KG)	0.8690	0.7292	0.8170	0.6829	0.9555	0.8781	0.8551	0.9161
CSK-DDI-MG (w/o Molecular Graph)	0.9023	0.7647	0.8186	0.7342	0.9638	0.9065	0.8910	0.9289
**CSK-DDI(Ours)**	**0.9114**	**0.7864**	**0.8654**	**0.8184**	**0.9716**	**0.9117**	**0.8975**	**0.9359**

a The bold corresponds to the best performance for each metric.

These results demonstrate the effectiveness of the proposed model. Specifically, (i) SSI-DDI, DeepDDI, DDI-Hybrid, and MI-DDI showed limited performance, as they rely solely on internal molecular representations without incorporating system-level biological context; (ii) KG-DDI consistently underperformed by leveraging macroscopic network embeddings while neglecting fine-grained topological features from molecular graphs; (iii) MUFFIN attempted to fuse molecular structures with KG semantics, but its performance fluctuated across datasets, suggesting limited robustness; and (iv) GoGNN and MRCGNN achieved competitive results by modeling hierarchical or dual-view relational structures, yet their global context remained restricted to the DDI network rather than a broader biomedical KG. Compared with all baselines, our model integrates complementary representations from motif sequences, molecular graphs, and external KG semantics, achieving the most robust performance across both datasets.

### 3.4 Inductive setting

In real-world drug discovery, newly approved drugs often lack sufficient interaction records, making it necessary to predict DDIs under limited supervision. To evaluate generalization to unseen drugs, we adopt an inductive setting based on a predefined partition of the drug set into seen drugs ($D_{\text{seen}}$) and unseen drugs ($D_{\text{unseen}}$), where $D_{\text{seen}}\cup D_{\text{unseen}}=D$ and $D_{\text{seen}}\cap D_{\text{unseen}}=\emptyset$. The model is trained only on drug pairs from $D_{\text{seen}}$, with no interaction labels involving $D_{\text{unseen}}$ used during training. We evaluate two cold-start scenarios:

- **S1 (semi-inductive):** drug pairs where exactly one drug is unseen, evaluating whether the model can transfer learned patterns to partially unseen inputs.
- **S2 (fully inductive):** drug pairs where both drugs are unseen ($d_{i},d_{j}\in D_{\text{unseen}}$), representing a more challenging generalization scenario.

For all drugs, representations are extracted via pre-trained feature encoders from three modalities: motif sequences, molecular graphs, and local KG subgraph structures, supplemented by molecular fingerprints. For unseen drugs absent from the DDI training data, although their interaction labels are missing, we leverage their contextual background within the KG for feature extraction, without introducing interaction label information. To align these multimodal representations into a unified semantic space, we introduce an auxiliary alignment objective inspired by TransE, serving as a regularization term during training:


(14) $$L_{\text{align}}=||e_{d_{i}}+r_{\text{type}}-e_{d_{j}}|{|}_{2}$$

where $r_{\text{type}}$ denotes the embedding of the corresponding DDI relation type. This constraint encourages all drug embeddings to follow the translational rules defined by $r_{\text{type}}$, ensuring the learned representations preserve the underlying relational structure and improve generalization to unseen drugs during inference.

The overall training objective combines this alignment loss with the main classification loss:


(15) $$L=L_{\text{DDI}}+\lambda L_{\text{align}}$$

where $L_{\text{DDI}}$ is the cross-entropy loss for DDI type prediction, and λ is a weighting coefficient empirically set to 0.1.

This geometric alignment ensures unseen drugs respect learned interaction patterns. As shown in Table 3, CSK-DDI significantly outperforms baselines in both scenarios. For S1, our model achieves a Macro-F1 of 50.3%, which improves upon CSMDDI by 4.8%. The advantage is further amplified in the challenging S2 setting, where we obtain 23.8% Macro-F1 and 39.5% Accuracy, outperforming the best baseline by 4.0% and 2.2%, respectively. This demonstrates highly effective inductive generalization.

**Table 3:** Performance evaluation in the inductive setting.^a^

Method	S1 partition		S2 partition	
	Macro-F1	ACC	Macro-F1	ACC
CSMDDI	45.5	62.6	19.8	37.3
HIN-DDI	37.3	8.9	8.8	27.6
KG-DDI	26.1	46.7	1.1	32.2
DeepLGF	39.7	60.7	4.8	31.9
**CSK-DDI**	**50.3**	**63.1**	**23.8**	**39.5**

a The bold corresponds to the best performance for each metric.

### 3.5 Ablation study

To investigate the contribution of different modalities within our framework, we compare the full CSK-DDI model with three ablation variants: a motif-only model, a model without the KG, and a model without the molecular graph.

As illustrated in Fig. 2, the full CSK-DDI model consistently achieves the best performance across all metrics on both Deng’s and Ryu’s datasets, indicating that the integration of these three modalities is essential for optimal prediction.

**Figure 2:**
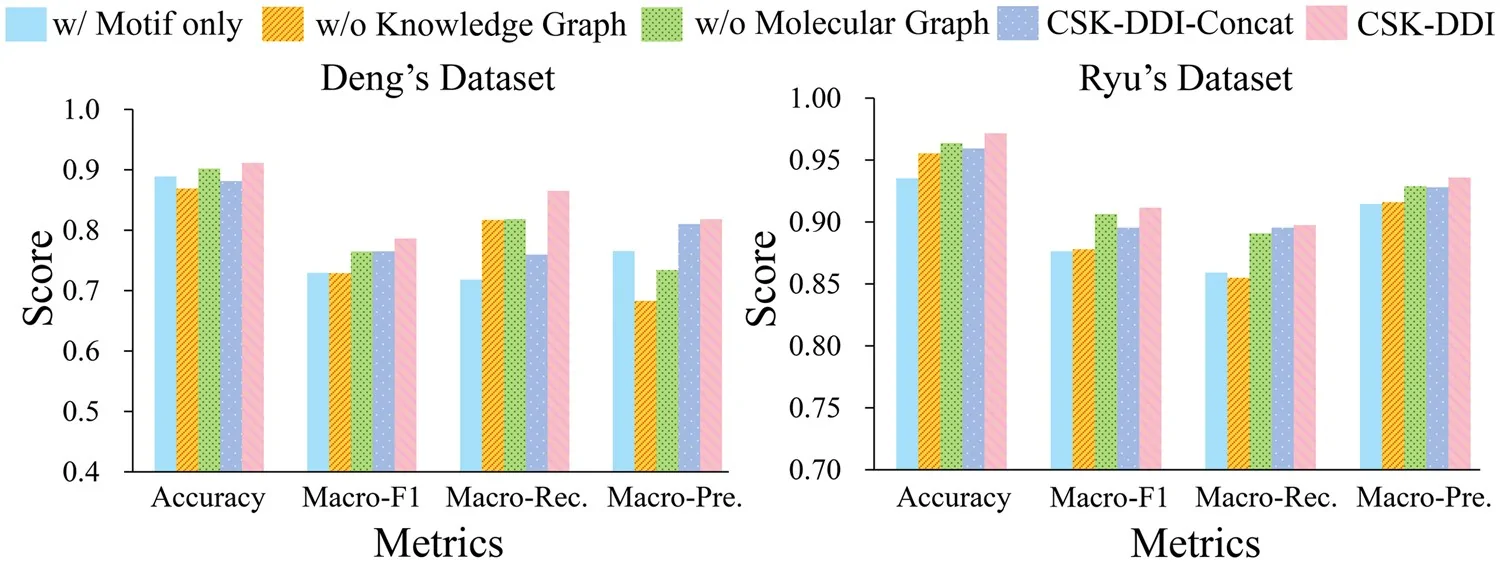
Performance comparison of the CSK-DDI framework against its ablation variants on Deng’s and Ryu’s datasets.

Specifically, removing the external KG leads to a noticeable performance degradation. On Deng’s dataset, the Macro-F1 drops by 5.72% and Accuracy decreases by 4.24%. This highlights the usefulness of macroscopic semantic relationships provided by the KG. Similarly, omitting the atomic-level molecular graph degrades the Macro-F1 by 2.17% and Accuracy by 0.91% on Deng’s dataset, suggesting that fine-grained topological structures provide necessary complementary information. Furthermore, relying solely on sequence motifs yields suboptimal results across both datasets. Beyond the choice of modalities, the fusion mechanism itself also matters. Replacing cross-attention with simple concatenation leads to a performance drop, confirming the benefit of the residual cross-modal design.

Overall, the performance drops observed in these variants confirm that both the three modalities and the cross-attention fusion mechanism contribute positively to the final DDI prediction.

### 3.6 Parameter sensitivity analysis

We analyze the sensitivity of CSK-DDI to two key hyperparameters on the Deng’s dataset: the learning rate (*lr*) and WD. Specifically, we evaluate combinations of lr∈1e–4,5e–4,1e–3,2e–3,5e–3 and WD∈1e–3,1e–4,1e–5,1e–6.

As shown in Fig. 3, the model performance varies across different parameter settings. Only Macro-F1 is reported here, with both Macro-F1 and accuracy results provided in Fig. 1, available as supplementary data at *Bioinformatics* online. The best result is achieved when lr=5e–4 and WD=1e–6, yielding a Macro-F1 score of 0.8436. This indicates that a moderate learning rate combined with a small WD provides a good balance between optimization and regularization.

**Figure 3:**
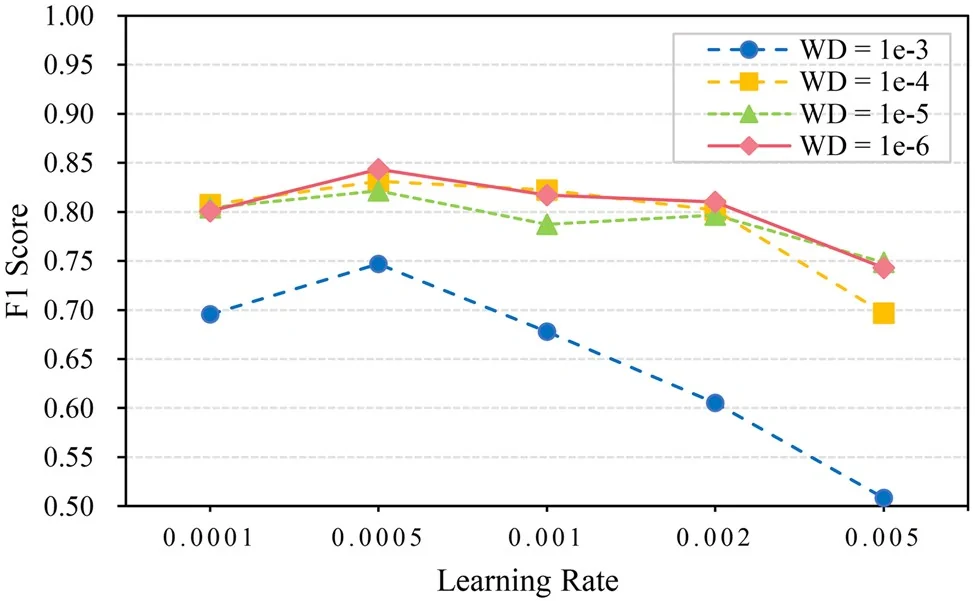
Hyper-parameter sensitivity analysis.

In contrast, larger learning rates lead to noticeable performance degradation. For example, when lr=5e–3 and WD=1e–3, the Macro-F1 score drops to 0.5085. These results highlight the importance of proper hyperparameter selection for achieving stable and effective DDI prediction.

### 3.7 Case study

In this section, we present a case study to examine the interpretability of CSK-DDI. We analyze the interaction between simvastatin, a cholesterol-lowering statin, and ketoconazole, an antifungal agent. The model assigns a high probability to a DDI type indicating that ketoconazole may increase the serum concentration and toxicity of simvastatin. As shown in Fig. 4, the model integrates atomic-level structural features with pathway-level biological context to provide complementary evidence.

**Figure 4:**
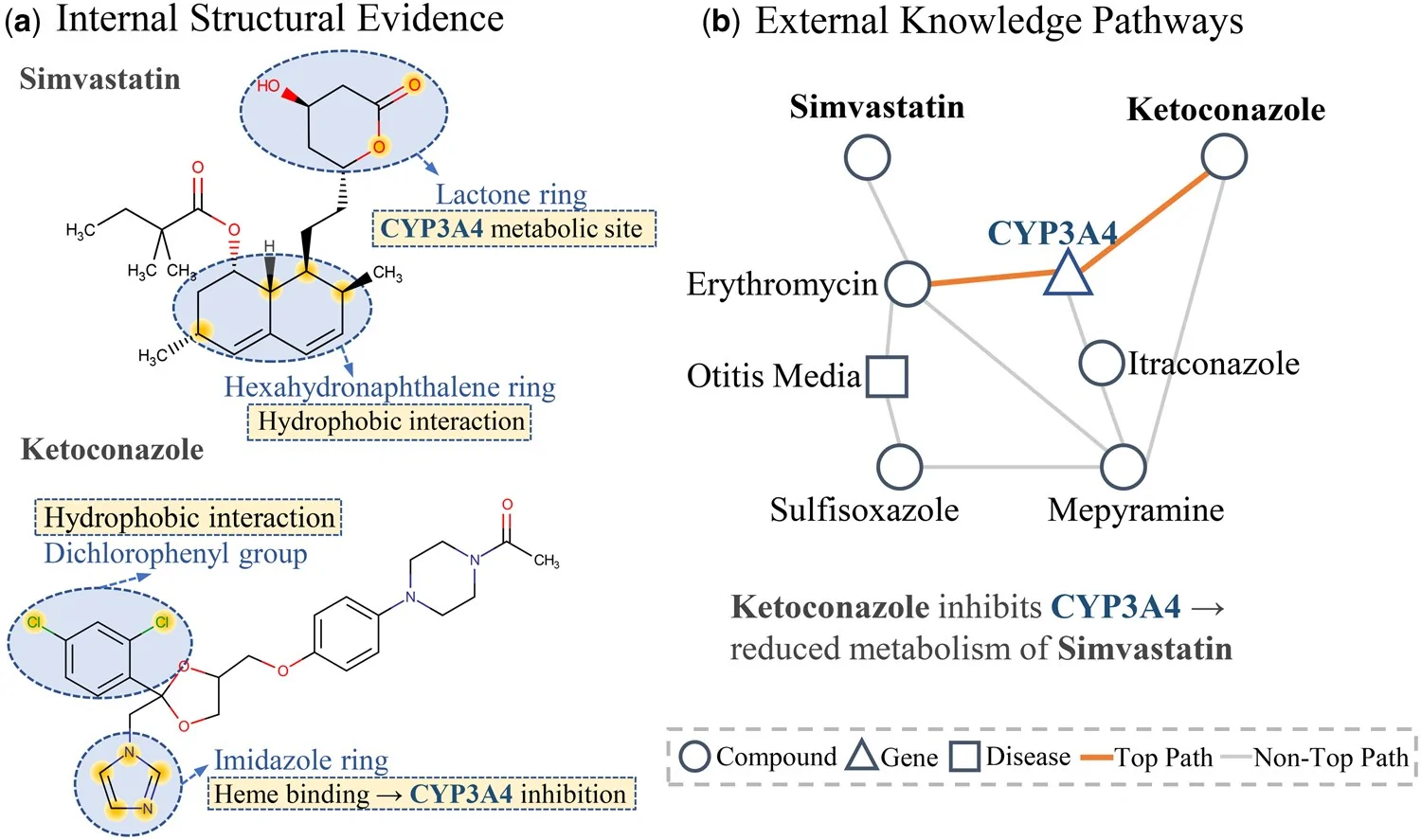
Visualization of dual-level evidence for the simvastatin-ketoconazole interaction. The model highlights relevant chemical substructures and metabolic pathways, suggesting a potential mechanism for the observed interaction.

At the structural level, attention visualization indicates that the model focuses on the lactone ring of simvastatin and the imidazole nitrogen of ketoconazole, both of which are functionally relevant for enzymatic metabolism and enzyme coordination.

At the KG level, top-weighted paths are predominantly associated with CYP3A4, identifying it as a metabolic bottleneck connecting the two drugs. Notably, these paths also involve erythromycin, another well-known CYP3A4 inhibitor, suggesting that the model captures broader pharmacological context beyond the target drug pair.

By integrating these two modalities, CSK-DDI proposes a plausible pharmacokinetic mechanism. Specifically, the imidazole nitrogen of ketoconazole may inhibit CYP3A4 activity, thereby reducing the metabolism of simvastatin via its lactone ring. This inhibition can increase systemic exposure to simvastatin, which has been associated with adverse effects such as myopathy and rhabdomyolysis. This predicted mechanism aligns with the reported interaction between the two drugs in DrugBank. Furthermore, an additional case study analyzing the interaction between carbachol and neostigmine is provided in the supplementary data. Overall, this case study demonstrates that CSK-DDI can identify high-risk drug combinations while providing interpretable evidence.

## 4 Conclusion

We propose CSK-DDI, a multi-modal framework that integrates functional motifs, molecular graphs, and external KGs for DDI prediction. Experimental results across benchmark datasets demonstrate that CSK-DDI consistently outperforms state-of-the-art baselines. Ablation studies further confirm that fusing these diverse modalities is essential for optimal predictive performance.

Beyond standard settings, CSK-DDI exhibits robust generalization in cold-start scenarios and provides mechanistic interpretability by aligning atomic attention with biological paths. Future research will aim to refine the attention mechanism to provide more intuitive and clearer biological explanations for drug interactions.

## Supplementary Material

btag800_Supplementary_Data

## Data Availability

All data used in this study are publicly available. DrugBank can be accessed at https://go.drugbank.com/releases/latest, DRKG at https://github.com/gnn4dr/DRKG, and the datasets from Deng and Ryu at https://github.com/Zhankun-Xiong/MRCGNN. The code for CSK-DDI is available at https://github.com/cgwcbz2/CSK-DDI and on Zenodo at https://doi.org/10.5281/zenodo.21415769.
