# Supplementary material for: CSK-DDI: compensating structural incompleteness with knowledge graph world knowledge for drug–drug interaction modeling
Source: Bioinformatics. 2026 Oct 28;42(11):btag800. doi: 10.1093/bioinformatics/btag800 (PMC13755760; doi:10.1093/bioinformatics/btag800)
Supplement: Supplementary file 1 [file btag800_supplementary_data.pdf]

Supplementary Materials

1 Training Setting Details

Our model is implemented with PyTorch 1.10.0. All code was developed on a Linux server equipped with 1 GPU (NVIDIA GeForce RTX 4090, 24GB), CUDA 11.3, and 16 vCPUs (Intel(R) Xeon(R) Gold 6430). Table S1 demonstrates all the hyperparameters of CSK-DDI.

Table S1. Hyperparameter Configuration for CSK-DDI

**Hyperparameter**	**Description**	**Value**
lr	Learning rate	0.001
weight_decay	Weight decay for Adam optimizer	1×10^{-4}
batch_size	Input batch size	128
epochs	Number of training epochs	80
hidden_dim	Hidden dimension for feature extractor	128
dim	Embedding dimension in fusion module	128
fusion_hidden	Hidden layer size in fusion classifier	512
num_heads	Number of attention heads in fusion module	4
dropout	Dropout rate in attention layers	0.2
gt_layer_num	Number of GT layers in feature extractor	4
gt_num_heads	Number of heads in GT layers	8
num_classes	Number of DDI relation types	65/86
extractor	Subgraph extraction method	randomWalk
num_paths	Number of random walk paths per drug	1
path_length	Length of each random walk path	32

2 Inductive Setting Details

To provide a more granular breakdown of the inductive setting described in the main text, we detail the construction of the training and test sets. Recall that the complete set of drugs D is partitioned into disjoint subsets of seen drugs (D_{seen}) and unseen drugs (D_{unseen}).

Let E denote the complete set of all DDI triplets (d_i, d_j, r) in our dataset. Based on the drug-level split ($D = D_{\text{seen}} \cup D_{\text{unseen}}$ and $D_{\text{seen}} \cap D_{\text{unseen}} = \emptyset$), the interaction datasets are constructed as follows:

- **Training Set (E_{train}):** Interactions exclusively between seen drugs. The model is trained solely on this subset:

$$E_{\text{train}} = \{(d_i, d_j, r) \in E \mid d_i \in D_{\text{seen}} \wedge d_j \in D_{\text{seen}}\}.$$

- **Test Set 1 (DS_1):** Interactions between two unseen drugs, evaluating the model's ability to generalize to entirely novel drug pairs (fully inductive scenario):

$$DS_1 = \{(d_i, d_j, r) \in E \mid d_i \in D_{\text{unseen}} \wedge d_j \in D_{\text{unseen}}\}.$$

- **Test Set 2 (DS_2):** Interactions between one seen and one unseen drug, assessing the model's ability to transfer knowledge from known drugs to partially novel pairs (partially inductive scenario):

$$DS_2 = \{(d_i, d_j, r) \in E \mid (d_i \in D_{\text{seen}} \wedge d_j \in D_{\text{unseen}}) \vee (d_i \in D_{\text{unseen}} \wedge d_j \in D_{\text{seen}})\}.$$

As established, the model is trained exclusively on E_{train} and evaluated on both DS_1 and DS_2 to comprehensively assess its generalization capabilities in cold-start scenarios.

3 Hyperparameter Results Details

During model training, the learning rate (lr) and weight decay (WD) are critical hyperparameters. The learning rate controls the step size for updating model parameters, while weight decay acts as a regularization technique to prevent overfitting. We evaluated the model primarily using Macro-F1 and Accuracy. The comprehensive results are presented in Figure S1.

To identify the best combination of learning rate and weight decay, we conducted extensive experiments. We tested various combinations, using learning rates of {1e-4, 5e-4, 1e-3, 2e-3, 5e-3} and weight decay values of {1e-3, 1e-4, 1e-5, 1e-6}. The results showed that CSK-DDI performed best with a learning rate of 5e-4 and a weight decay of 1e-6, achieving a peak Macro-F1 score of 0.8436. This indicates that this combination optimally balances learning and regularization, leading to improved prediction accuracy.

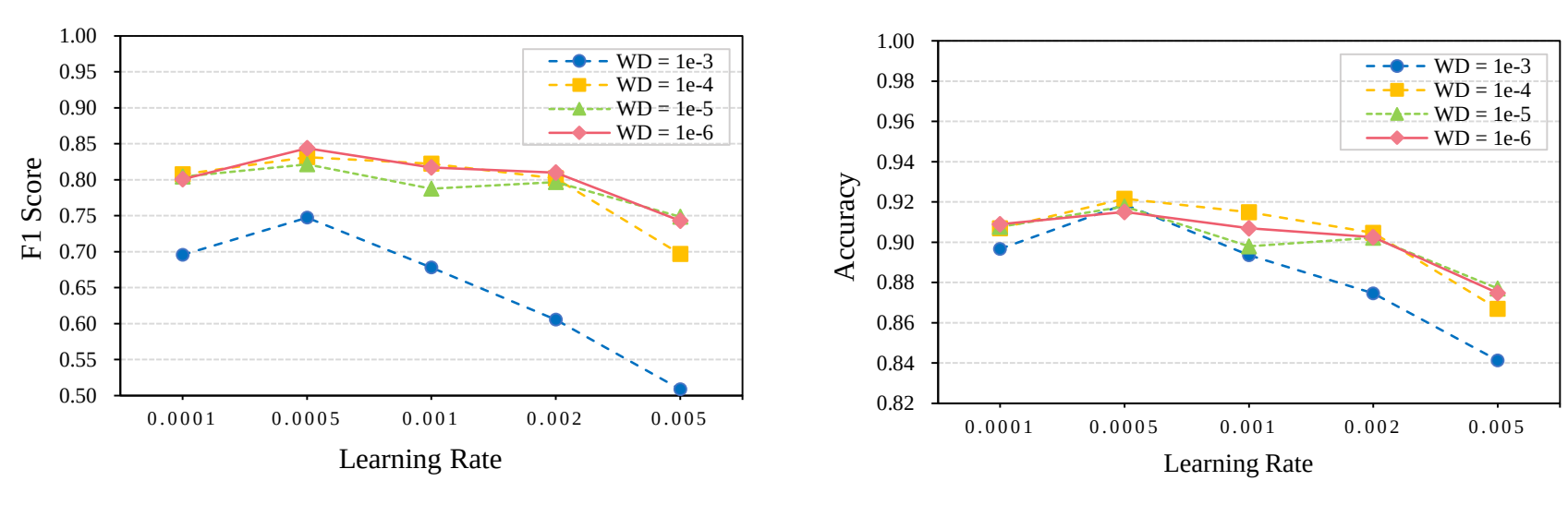

Figure S1. Performance variations of CSK-DDI across different combinations of learning rate and weight decay.

The choice of these hyperparameters significantly impacts the model's convergence speed and generalization ability. A high learning rate (e.g., $lr \geq$ 2e-3) can cause training instability and a sharp decline in performance, while a suitable rate ensures stable

optimization. Similarly, too much weight decay (e.g., WD = 1e-3) can cause underfitting and restrict performance across all learning rates, while too little may not adequately prevent overfitting.

4 Motif Length and Truncation

To optimize computational efficiency while preserving the structural information of molecules, we analyzed the length distribution of all extracted motifs in the dataset. As illustrated in Figure S2, the motif lengths exhibit a clear long-tailed distribution. The majority of motifs are relatively short, with the peak frequency occurring in the length range of 5–9 (894 motifs).

Based on this empirical distribution, we set the motif sequence truncation length to 26 in our implementation. Statistical analysis reveals that 98.7% of the extracted motifs (1,709 out of 1,731) have a length of 25 or less. Only a negligible fraction (approximately 1.3%) of outlier motifs exceed this length. By truncating sequences at a maximum length of 26, our model effectively avoids the excessive memory consumption and computational overhead associated with zero-padding matrices to accommodate rare extreme outliers (e.g., the single motif with a length ≥ 100). This strategy ensures high computational efficiency without sacrificing meaningful structural representations.

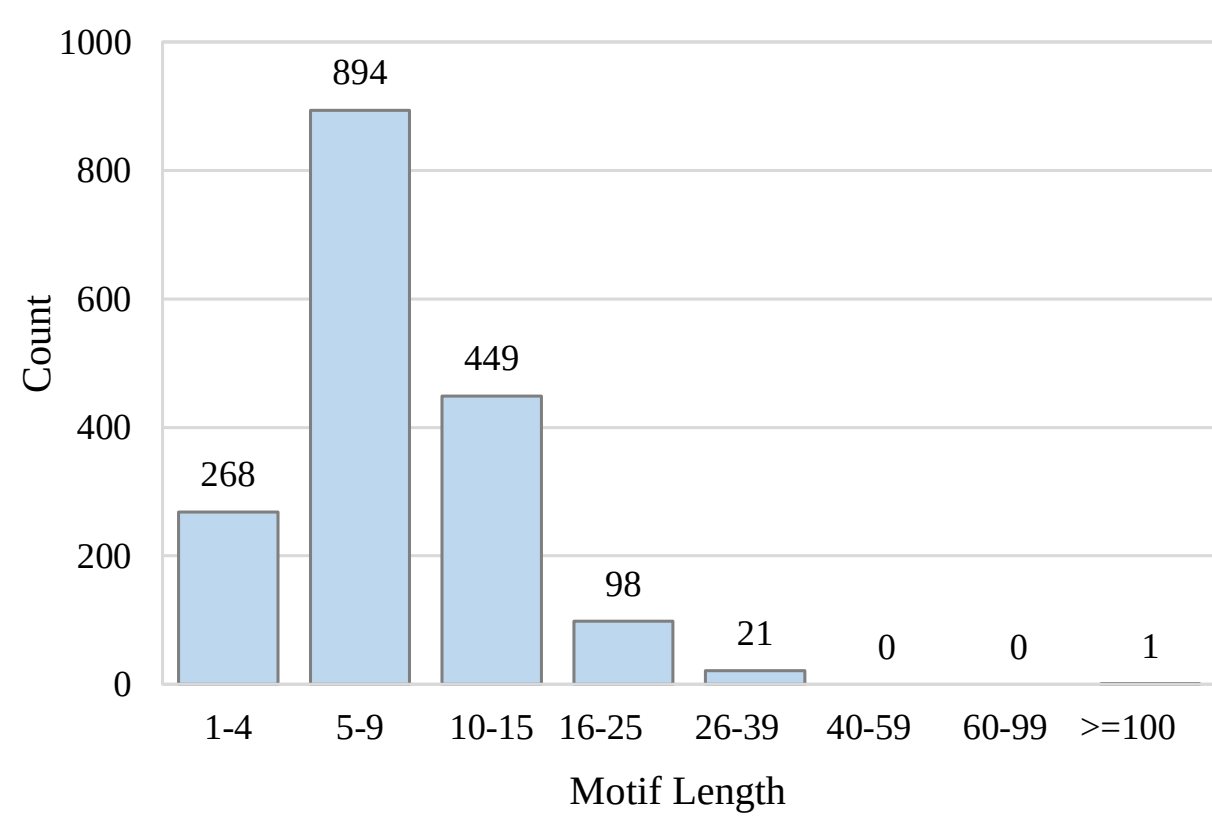

Figure S2. Distribution of extracted motif lengths across the dataset. The truncation threshold is set at 26.

5 Case Study II: carbachol and neostigmine

In this supplementary section, we present an additional case study to further explore the interpretability of the proposed CSK-DDI model. We analyze the interaction

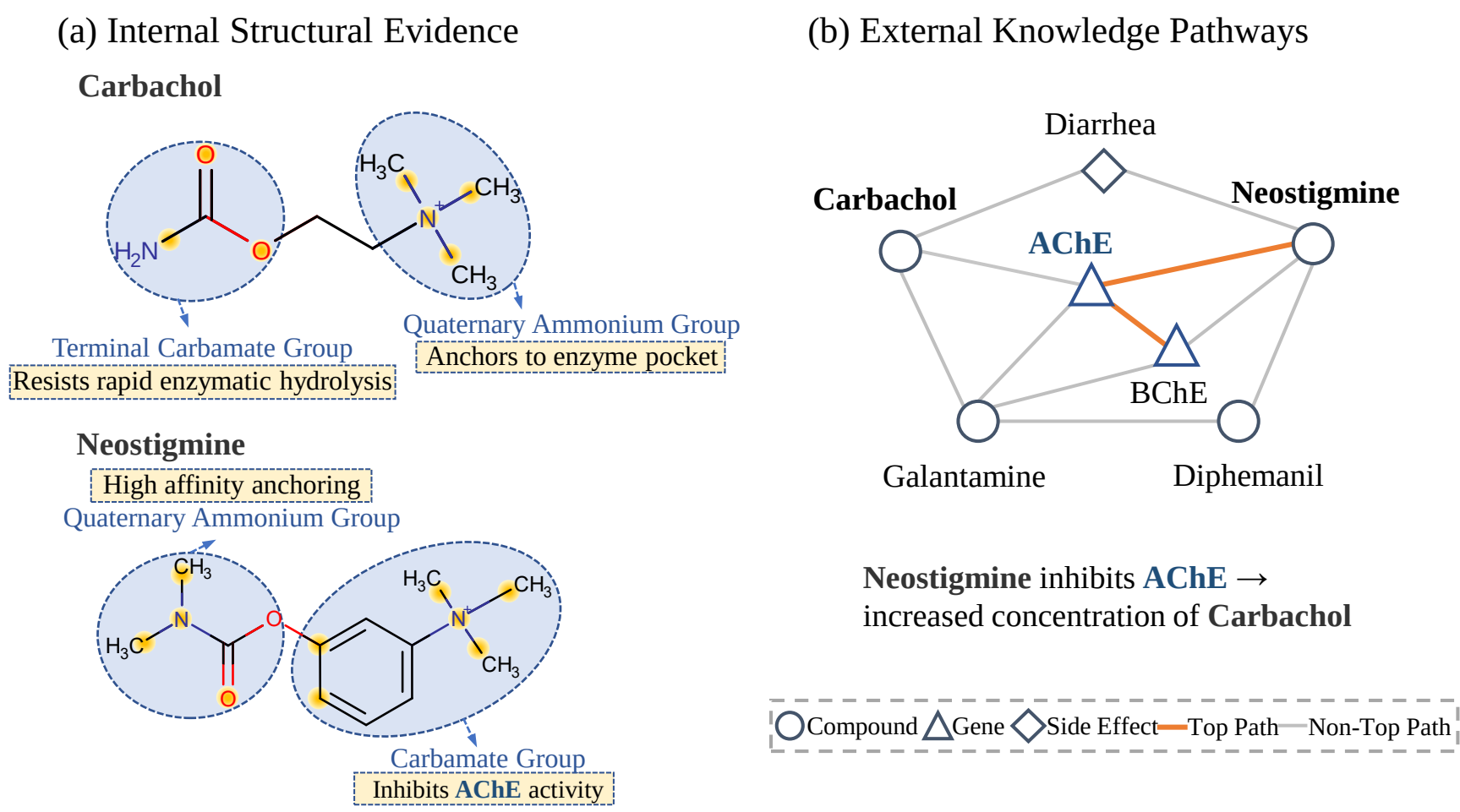

Figure S3. Visualization of dual-level evidence for the carbachol-neostigmine interaction.

between carbachol, a cholinergic agonist, and neostigmine, a reversible cholinesterase inhibitor. The model predicts a high probability for a DDI type indicating that neostigmine may increase the serum concentration and cholinergic toxicity of carbachol. As illustrated in Figure S3, CSK-DDI effectively integrates atomic-level molecular features with pathway-level biological knowledge to provide insights into the underlying mechanism.

At the structural level, attention visualizations on the molecular graphs reveal that the model concentrates on specific, pharmacologically relevant functional groups. For carbachol, the model assigns higher attention weights to the oxygen and nitrogen atoms within its terminal carbamate group, as well as its quaternary ammonium group. Similarly, for neostigmine, the attention is largely focused on the ester oxygen of its carbamate moiety and the nitrogen of its quaternary ammonium group. These regions are closely associated with their mechanism of action: the positively charged quaternary ammonium groups facilitate the anchoring of the molecules to the anionic site of the AChE enzyme, while the carbamate groups interact directly with its esteratic site.

At the external knowledge level, the KG summarization module identifies a concise set of critical sub-graphs connecting the two drugs. The top-weighted paths are predominantly associated with Acetylcholinesterase (AChE), highlighting its potential role as the primary competitive target. Notably, the model captures relational paths suggesting that the inhibition of AChE by neostigmine impedes the breakdown of cholinergic agonists, thereby potentially increasing carbachol concentration. Furthermore, the extracted biological context includes other relevant agents like Galantamine and Diphemanil, demonstrating that the model leverages a broader pharmacological network to support the predicted interaction beyond just the target drug pair.

By synthesizing these dual-level modalities, CSK-DDI proposes a reasonable mech-

anism for this interaction. At the pharmacodynamic level, neostigmine inhibits AChE activity, reducing the breakdown of acetylcholine at cholinergic synapses, while carbachol simultaneously acts as a direct agonist at the same receptors. This synergistic amplification of cholinergic signaling likely represents the primary driver of the observed toxicity, potentially manifesting as adverse effects such as diarrhea. At the pharmacokinetic level, while carbachol's carbamate structure already confers inherent resistance to enzymatic hydrolysis, concurrent AChE inhibition by neostigmine may further limit its clearance to some extent, though the magnitude of this contribution remains uncertain. Overall, this supplementary case indicates that CSK-DDI can identify high-risk interactions while offering biologically meaningful interpretations.
